# Supplementary material for: Trafficking of the glutamate transporter is impaired in LRRK2-related Parkinson’s disease
Source: Acta Neuropathol. 2022 May 21;144(1):81–106. doi: 10.1007/s00401-022-02437-0 (PMC9217889; doi:10.1007/s00401-022-02437-0)
Supplement: Supplementary file 2 — Supplementary file2 (DOCX 50016 KB) [file 401_2022_2437_MOESM2_ESM.docx]

**Supplementary materials and methods**

**Quantitative Polymerase Chain Reaction (qPCR***)*

Total RNA was extracted from mice striata or from primary striatal astrocytes with the Total RNA Purification kit (NORGEN Biotek) and quantified by absorbance in a NanoDrop 2000c UV-Vis spectrophotometer (ThermoFisher Scientific). cDNA was synthesized with the All-in-One Cdna Synthesis SuperMix (Bimake) following manufacturer’s instructions. Gene expression was quantified by qPCR in real-time PCR reactions with Sybr Green technology in a CFX96 Touch Real-Time PCR Detection System (Bio-Rad). 30 ng cDNA were used in iTaq Universal SYBR Green Supermix (Bio-Rad) at the following conditions: stage 1: 95 °C, 5 min; stage 2: 39 x (95 °C, 15 s; 60 °C, 30 s).

The primers were as follows: mSLC1A2 fw: GGTGGAAAGCCGGGACGTGGATTA; mSLC1A2 rev: GCTTGGGCATATTGTTGGCACCCT; SLC1A3 fw: ATCCGGGAGGAGATGGTGCCCGT; SLC1A3 rev: AGGATGCCCAGAGGCGCATACCACA; ACTIN fw: TACCACCATGTACCCAGGCATT; ACTIN rev: ACTCATCGTACTCCTGCTTGCTGA; mGAPDH fw: GAGAGTGTTTCCTCGTCCCG; mGAPDH rev: ACTGTGCCGTTGAATTTGCC; TRANSFERRIN RECEPTOR fw TATAAGCTTTGGGTGGGAGGCA; TRANSFERRIN RECEPTOR rev AGCAAGGCTAAACCGGGTGTATGA;

Primers for Slc1a2, Slc1a3, β-Actin and Transferrin were purchased from Sigma Aldrich while primers for GAPDH were purchased from Metabion International AG. The quantification of the gene relative expression was carried out by the Delta-Delta Ct method [4] by normalizing to the reference genes GAPDH, β-Actin and Transferrin receptor. A dissociation curve was built in the 60-95 °C range to confirm the specificity of the amplification product.

**Western blot on injected oocytes**

Western blot analysis on injected oocytes was performed as in [3]. Briefly, oocytes were homogenized in 20 mM HEPES solution (pH 7.3), supplemented with phosphatase and protease inhibitors. To remove the abundant yolk platelets, samples were centrifuged at 800g for 5 minutes RT. Then, protein concentration was measured using the Pierce® BCA Protein Assay Kit. Oocytes samples were resolved by electrophoresis on pre-cast 4–20 % tris-glycine polyacrylamide gels and transferred to polyvinylidene difluoride membranes using a semi-dry Biorad transfer machine with the 1X Transfer Buffer at 25 V for 20 min. Membranes were incubated in TBS-T plus 5 % skimmed milk for 1 h RT, and then incubated overnight with primary antibodies diluted in TBS-T plus 5 % skimmed milk. The following primary antibodies were used: a mouse anti-β-actin (A1978, Sigma-Aldrich, 1:10000) and a rabbit anti-LRRK2 (MJFF2 c41-2; Abcam, 1:300). Membranes were subsequently rinsed and incubated for 1h at RT with the appropriate HRP-conjugated secondary antibodies. The visualization of the signal was conducted using Immobilon® Forte Western HRP Substrate and the VWR® Imager Chemi Premium.

**Liquid-chromatography mass spectrometry (LC-MS) analysis**

Endogenous Glt-1 was immunoprecipitated from homogenized WT and LRRK2 G2019S mouse striata using 5 µg of a rabbit anti-Glt-1 antibody (Abcam; ab205248) in RIPA buffer (1.5 mg of total protein each). Magnetic beads (Protein A/G Magnetic Beads, BioTool) (50 µL) were used to pre-clear and precipitate the transporter. Beads were then washed three times using 1 ml of RIPA buffer and proteins loaded on pre-cast 4–20 % tris-glycine polyacrylamide gels (Biorad). Gel bands were excised, cut into small pieces and destained with a solution of 60 % NH_4_HCO_3_ 200 mM/40 % acetonitrile (ACN) at 37 °C. Disulfide bridges were reduced with 2 mM Tris (2-carboxyethyl)phosphine hydrochloride (TCEP) in 50 mM NH_4_HCO_3_ at 56 °C for 1 h, and cysteine residues were alkylated with 4 mM methyl methanethiosulfonate (MMTS) with 50 mM NH_4_HCO_3_ in the dark at room temperature for 45 min. Gel samples were washed twice with 50 mM NH_4_HCO_3_ and ACN alternatively and vacuum-dried. Samples were incubated with 12.5 ng/µL trypsin (Sequencing Grade Modified Trypsin, Promega) in 50 mM (NH_4_)_2_HCO_3_ and protein digestion was carried out overnight at 37 °C. Peptides were extracted with three changes of 50 % ACN/ 0.1 % formic acid (FA). Samples were vacuum-dried and stored at −20 °C. Samples were analyzed using a LTQ Orbitrap XL mass spectrometer (Thermo Fisher Scientific) coupled to a HPLC Ultimate 3000 (Dionex - Thermo Fisher Scientific) through a nanospray source (NSI), as described previously [1]. Samples were resuspended in 30 µL of 3 % ACN/0.1 % FA and each sample was acquired twice. Raw data files were analyzed with MaxQuant [2] software (v. 1.5.1.2) interfaced with Andromeda search engine. Protein search was performed against the mouse section of the UniProt database (*Mus musculus*, version 2020-09-30, 55494 entries). Enzyme specificity was set to trypsin with up to one missed cleavage allowed, while methylthio-cysteine and oxidized methionine were set as fixed and variable modifications, respectively. A minimum of two peptides was required for protein identification, and a false discovery rate (FDR) < 0.01, both at the peptide and protein level, was used to filter the results. To estimate the relative protein abundance across samples, the intensity values calculated by the software were used. The datasets were compared to highlight significant differences in the protein abundances: common and unique Glt-1 binders between the LRRK2 WT and G2019S genetic environments were identified performing a Z-test on the log-transformed values of the IP/CTRL ratios and considering only proteins with p < 0.05 and Fold Changes (FC) > 3.5. Gene Ontology (GO) terms were employed to identify discrete functional enrichments in order to extract information about novel networks of interactors in relationship to the different subcellular localization of the transporter. G:profiler (https://biit.cs.ut.ee/gprofiler/gost) was used for enrichment analysis. Glt-1-related protein networks in LRRK2 G2019S pathogenic background were ranked based on the fold-change affinity over the control (>3.5).

**Bibliography**

1. Battisti I, Ebinezer LB, Lomolino G, Masi A, Arrigoni G (2021) Protein profile of commercial soybean milks analyzed by label-free quantitative proteomics. Food Chem 352:129299. doi: 10.1016/j.foodchem.2021.129299

2. Cox J, Mann M (2008) MaxQuant enables high peptide identification rates, individualized p.p.b.-range mass accuracies and proteome-wide protein quantification. Nat Biotechnol 26:1367–1372. doi: 10.1038/nbt.1511

3. Lin-Moshier Y, Marchant JS (2013) A rapid Western blotting protocol for the Xenopus oocyte. Cold Spring Harb Protoc 2013. doi: 10.1101/pdb.prot072793

4. Livak KJ, Schmittgen TD (2001) Analysis of relative gene expression data using real-time quantitative PCR and the 2(-Delta Delta C(T)) Method. Methods 25:402–408. doi: 10.1006/METH.2001.1262

**Supplementary Figure 1**

**
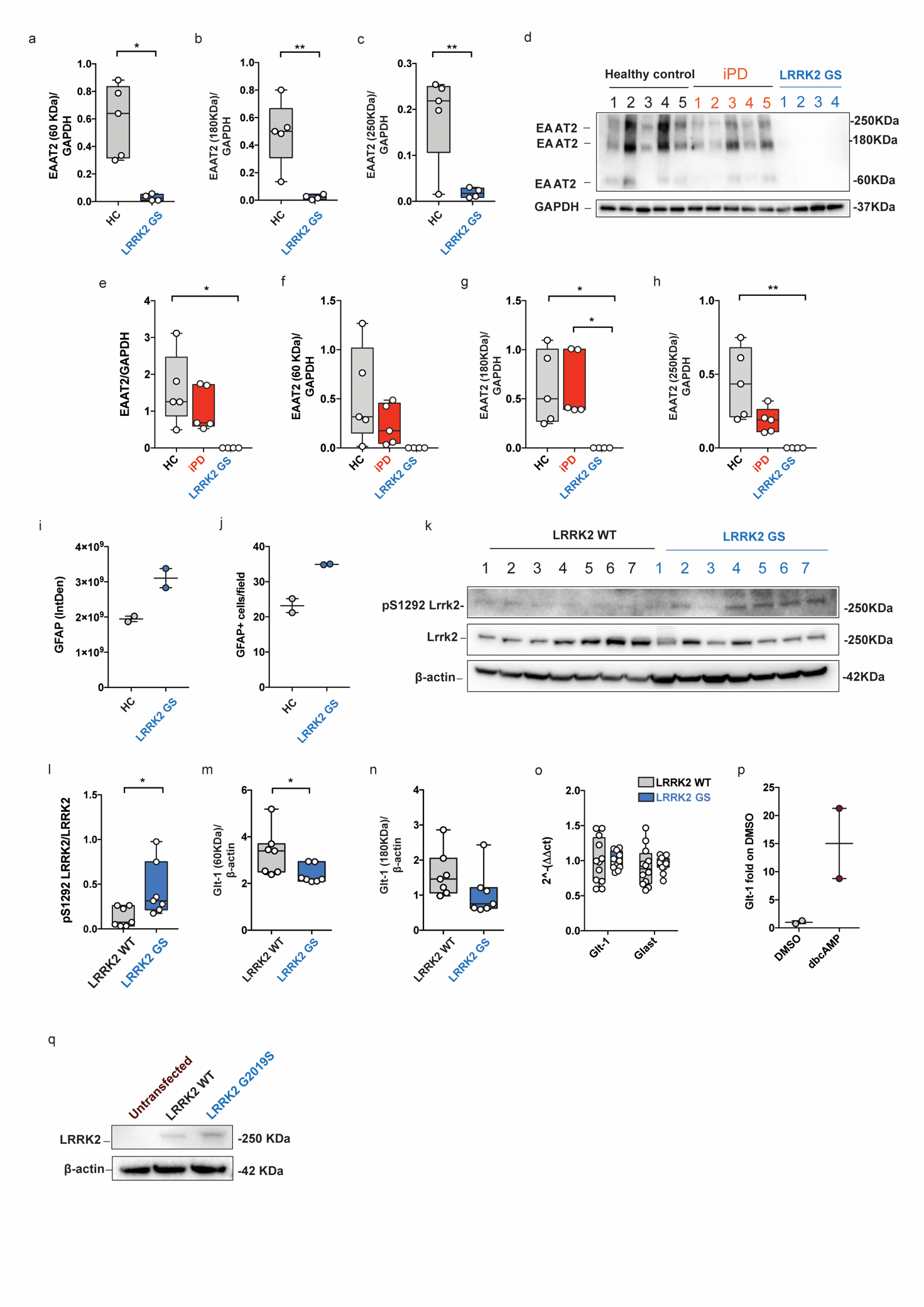
**

**Supplementary Figure 1**

a-c) Western blot quantification of the monomeric fractions (60 KDa) as well as of the multimeric fractions (180 KDa and 250 KDa) of EAAT2 in LRRK2 G2019S PD patients (n=4) compared to healthy controls (n=5); d) Western blot analysis of healthy controls, idiopathic PD patients (iPD) and LRRK2 G2019S caudate and putamen lysates using an anti-EAAT2 antibody; e-h) Relative quantification of the total EAAT2 as well as of the monomeric fractions (60 KDa) and multimeric fractions (180 KDa and 250 KDa) of EAAT2 in the healthy controls (n=5), iPD patients (n=5) and LRRK2 G2019S PD patients (n=4). The band intensity was performed using ImageJ and normalized to the housekeeping protein GAPDH; i-j) Quantification of GFAP IntDen and of GFAP^+^ positive cells in the caudate and putamen of healthy controls (n=2) and LRRK2 G2019S PD patients (n=2); k) Western blot analysis of LRRK2 WT and LRRK2 G2019S striatal lysates blotted with the following antibodies: anti-pS1292 LRRK2, anti-total LRRK2 and the housekeeping β-actin; l) Quantification of Ser1292 phosphorylation (pS1292 LRRK2/total LRRK2) was performed using ImageJ (n=7 striatal samples for both LRRK2 WT and LRRK2 G2019S 4-month old mice; m-n) Quantification of the 60 KDa and 180 KDa Glt-1 bands in LRRK2 WT and LRRK2 G2019S mice; n=7 animals for each genotype; o) qPCR analysis of Glt-1 and Glast mRNA in the striatum of LRRK2 WT and LRRK2 G2019S mice; n=12 animals for each genotype; p) qPCR analysis of Glt-1 mRNA in primary LRRK2 WT striatal astrocytes untreated of treated with dbcAMP for 10 days; experiment performed twice on n=2 independent cell cultures; q) Western blot analysis of oocytes co-injected with the mRNA of the excitatory amino acid transporter 2 and the mRNA of the human LRRK2 WT or G2019S, blotted with the antibodies against the total LRRK2 and the housekeeping β-actin. Statistical analysis was performed in a-c, o using Unpaired T-test and in k-l using Mann-Whitney test. Statistical analysis was performed in e, g using Kruskal-Wallis test (e: P=0.006; g: P=0.007) followed by Dunn's multiple comparisons test and in f, h using One-way ANOVA test (f: F=3.07 and P=0.08; h: F=9.49 and P=0.004) followed by Tukey's multiple comparisons test.

**Supplementary Figure 2**

**_
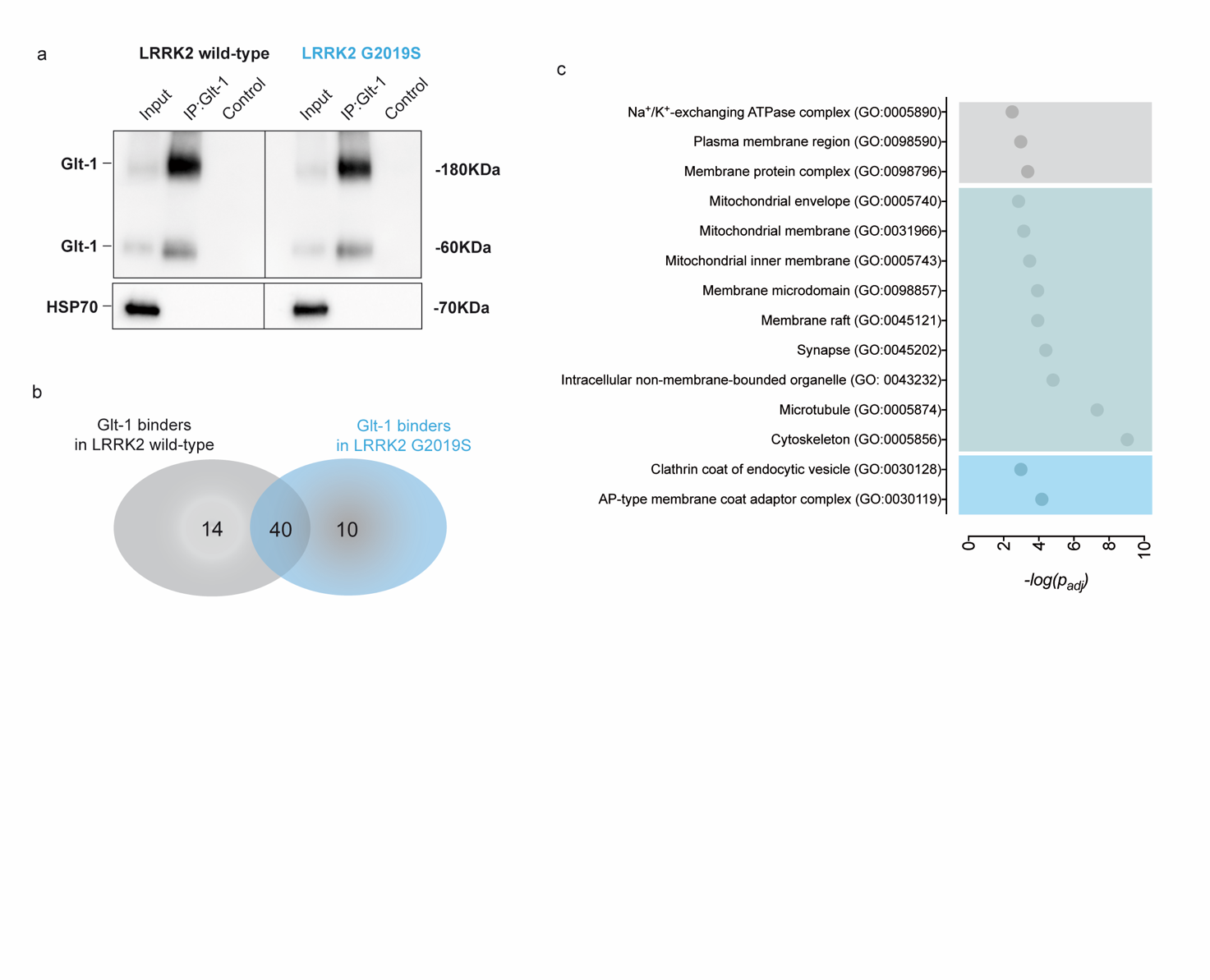
_**

**Supplementary Figure 2- Screening of Glt-1 protein-protein interactions**

a) Striatal Glt-1 immunoprecipitated from LRRK2 WT and G2019S brains was resolved by immunoblotting. Anti-HSP70 was applied to normalize for protein content; b) Venn diagram summary of interacting proteins, colored for different background (grey for LRRK2 WT, light blue for LRRK2 G2019S, dark blue for LRRK2 WT and G2019S shared interacting proteins); c) Selected GO term enrichments from CC categories using gProfiler were plotted using -log(P*adj*) values.

**Supplementary Figure 3**

**
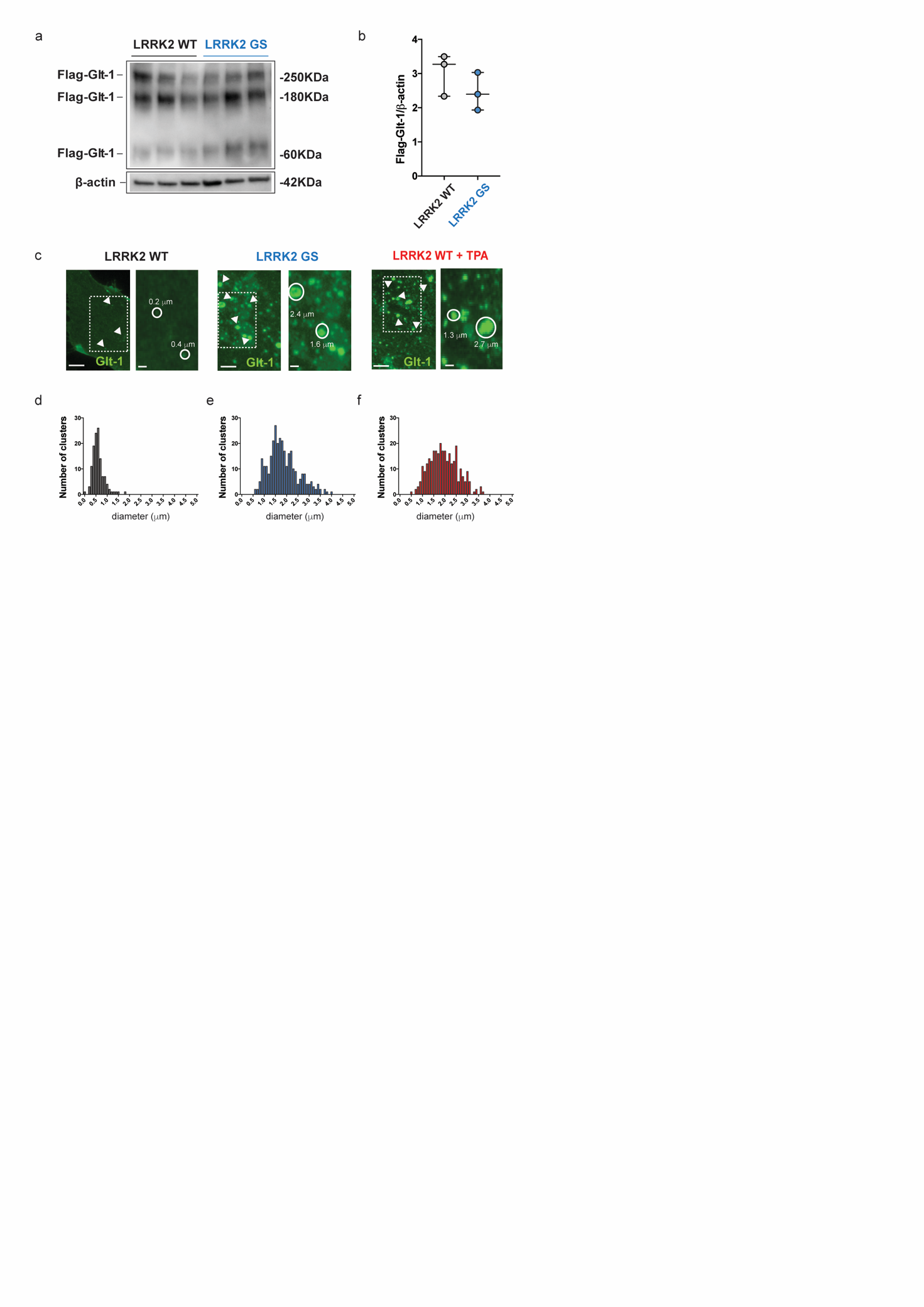
**

**Supplementary Figure 3**

a) Western blot analysis of transfected Flag-Glt-1 expression in primary striatal LRRK2 WT and G2019S astrocytes using an anti-Flag-HRP antibody; b) Relative quantification of band intensity was performed using ImageJ and normalized to β-actin; experiment performed in triple; c) Representative epifluorescence images of Glt-1 clusters in Flag-Glt-1 transfected primary striatal astrocytes from LRRK2 WT (untreated or treated with TPA) and from LRRK2 G2019S astrocytes. Scale bars 5 μm; insets 1 μm. d) Frequency distribution of Glt-1 cluster diameter in untreated LRRK2 WT (grey bars); e) Frequency distribution of Glt-1 cluster diameter in LRRK2 G2019S (blue bars); f) Frequency distribution of Glt-1 cluster diameter in LRRK2 WT treated with TPA (d; red bars); n=5 cells analyzed for each group. Statistical analysis in b was performed using Mann-Whitney test.

**Supplementary Figure 4**

**
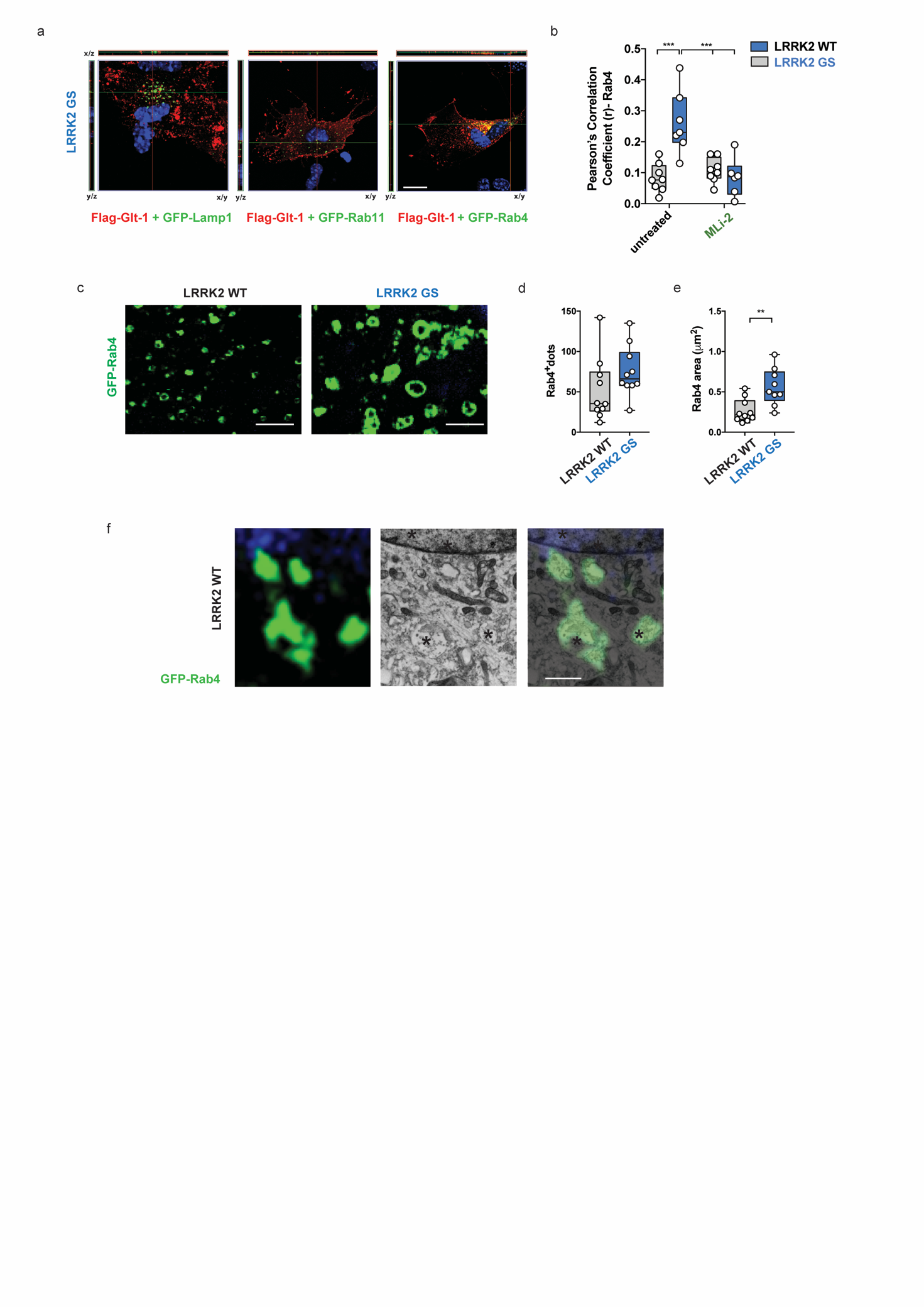
**

**Supplementary Figure 4**

a) Orthogonal z-stack projections of LRRK2 G2019S astrocytes co-transfected with Flag-Glt-1 (red) and GFP-Lamp1, GFP-Rab11 or GFP-Rab4 (green). Scale bar 20 μm; b) Quantitative analysis of the Pearson’s Correlation Coefficient of Glt-1 colocalizing with the Rab4-positive vesicles in LRRK2 WT and G2019S astrocytes under basal conditions or upon MLi-2 application; at least n=6 cells analyzed for each group; c) Representative confocal microscopy images of LRRK2 WT and G2019S primary striatal astrocytes transfected with GFP-Rab4 and Flag-Glt-1. Scale bars: 2 μm; d,e) Quantification of Rab4-positive vesicle number and area; at least n=9 cells analyzed for each group and experiments performed at least in triple; f) Representative confocal (left panel), electron microscopy (middle panel) and merge (right panel) image of a LRRK2 WT primary astrocyte transfected with Rab4-GFP (green). The image reveals the co-localization of early endosomal structures identified by transmission microscopy with the GFP-Rab4-positive vesicles identified by confocal microscopy; scale bar: 1 μm. Statistical analysis in b was performed using Two-way ANOVA test (Treatment F=15.79 and P=0.004; Genotypes F=16.38 and P =0.004) followed by Tukey's multiple comparisons test. Statistical analysis in d, e was performed using Unpaired t-tests.

**Supplementary Figure 5**


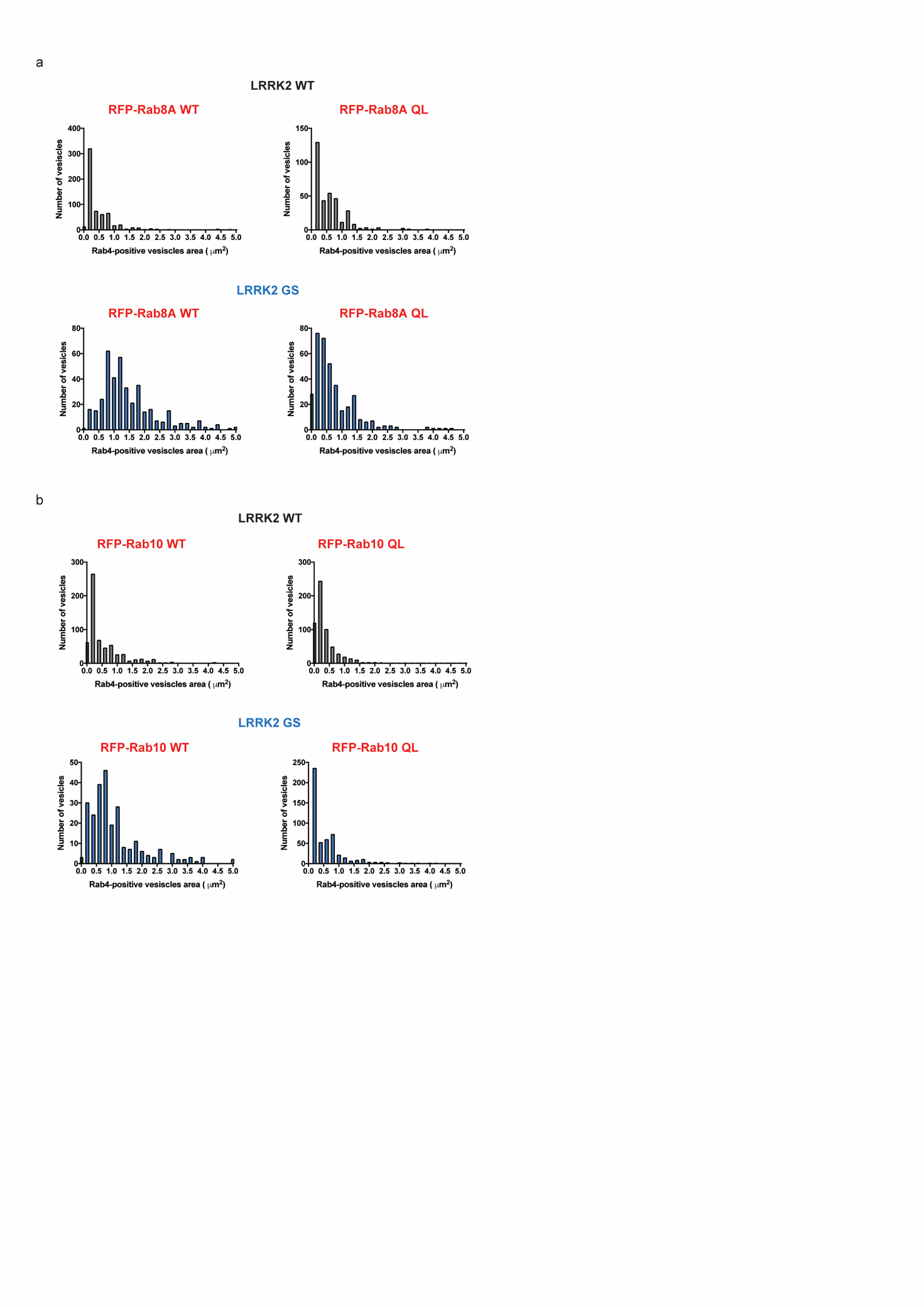


**Supplementary Figure 5**

a,b) Frequency distribution of Rab4-positive vesicles area in LRRK2 WT and LRRK2 G2019S astrocytes. At least n=6 cells analyzed for each group and experiments performed at least in triple.

**Supplementary Figure 6**


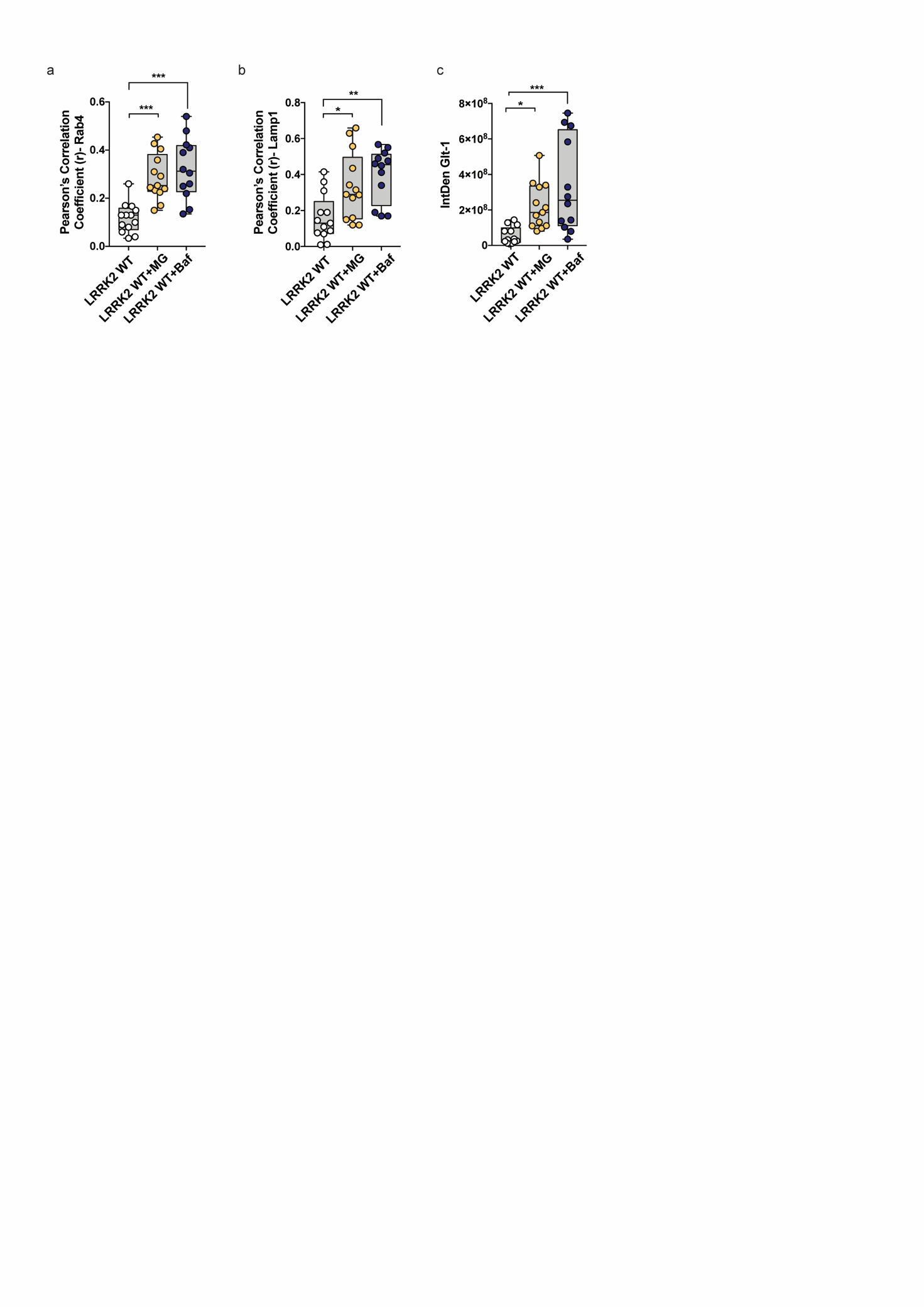


**Supplementary Figure 6**

a) Quantification of Glt-1 IntDen in the Rab4-positive vesicles in LRRK2 WT astrocytes under basal conditions (n=13 cells) or upon MG132 (n=13 cells) or Bafilomycin application (n=12 cells); b) Quantification of Glt-1 IntDen in the endogenous Lamp1-positive structures in LRRK2 WT astrocytes under basal conditions (n=13 cells) or upon MG132 (n=13 cells) or Bafilomycin (n=12 cells) application; experiments performed at least in triple; c) Quantitative analysis of Glt-1 IntDen in LRRK2 WT astrocytes, under basal condition or after application of MG132 or Bafilomycin A1 (n=13 cells for untreated LRRK2 WT, n=13 cells for LRRK2 WT+MG132 and n=12 LRRK2 WT+Bafilomycin; experiments performed at least in triple; Statistical analysis in a-c was performed using One-way ANOVA test (a: F=16.06 and P<0.0001; b: F=7.79 and P=0.001; c:F=8.76 and P=0.0008) followed by Tukey's multiple comparisons test.
