# Supplementary material for: Trafficking of the glutamate transporter is impaired in LRRK2-related Parkinson’s disease
Source: Acta Neuropathol. 2022 May 21;144(1):81–106. doi: 10.1007/s00401-022-02437-0 (PMC9217889; doi:10.1007/s00401-022-02437-0)
Supplement: Supplementary file 1 — Supplementary file1 (DOCX 50009 KB) [file 401_2022_2437_MOESM1_ESM.docx]

**Supplementary Figure 1**

**Supplementary Figure 1**

a-c) Western blot quantification of the monomeric fractions (60 KDa) as well as of the multimeric fractions (180 KDa and 250 KDa) of EAAT2 in LRRK2 G2019S PD patients (n=4) compared to healthy controls (n=5); d) Western blot analysis of healthy controls, idiopathic PD patients (iPD) and LRRK2 G2019S caudate and putamen lysates using an anti-EAAT2 antibody; e-h) Relative quantification of the total EAAT2 as well as of the monomeric fractions (60 KDa) and multimeric fractions (180 KDa and 250 KDa) of EAAT2 in the healthy controls (n=5), iPD patients (n=5) and LRRK2 G2019S PD patients (n=4). The band intensity was performed using ImageJ and normalized to the housekeeping protein GAPDH; i-j) Quantification of GFAP IntDen and of GFAP^+^ positive cells in the caudate and putamen of healthy controls (n=2) and LRRK2 G2019S PD patients (n=2); k) Western blot analysis of LRRK2 WT and LRRK2 G2019S striatal lysates blotted with the following antibodies: anti-pS1292 LRRK2, anti-total LRRK2 and the housekeeping β-actin; l) Quantification of Ser1292 phosphorylation (pS1292 LRRK2/total LRRK2) was performed using ImageJ (n=7 striatal samples for both LRRK2 WT and LRRK2 G2019S 4-month old mice; m-n) Quantification of the 60 KDa and 180 KDa Glt-1 bands in LRRK2 WT and LRRK2 G2019S mice; n=7 animals for each genotype; o) qPCR analysis of Glt-1 and Glast mRNA in the striatum of LRRK2 WT and LRRK2 G2019S mice; n=12 animals for each genotype; p) qPCR analysis of Glt-1 mRNA in primary LRRK2 WT striatal astrocytes untreated of treated with dbcAMP for 10 days; experiment performed twice on n=2 independent cell cultures; q) Western blot analysis of oocytes co-injected with the mRNA of the excitatory amino acid transporter 2 and the mRNA of the human LRRK2 WT or G2019S, blotted with the antibodies against the total LRRK2 and the housekeeping β-actin. Statistical analysis was performed in a-c, o using Unpaired T-test and in k-l using Mann-Whitney test. Statistical analysis was performed in e, g using Kruskal-Wallis test (e: P=0.006; g: P=0.007) followed by Dunn's multiple comparisons test and in f, h using One-way ANOVA test (f: F=3.07 and P=0.08; h: F=9.49 and P=0.004) followed by Tukey's multiple comparisons test.

**Supplementary Figure 2**

**Supplementary Figure 2- Screening of Glt-1 protein-protein interactions**

a) Striatal Glt-1 immunoprecipitated from LRRK2 WT and G2019S brains was resolved by immunoblotting. Anti-HSP70 was applied to normalize for protein content; b) Venn diagram summary of interacting proteins, colored for different background (grey for LRRK2 WT, light blue for LRRK2 G2019S, dark blue for LRRK2 WT and G2019S shared interacting proteins); c) Selected GO term enrichments from CC categories using gProfiler were plotted using -log(P*adj*) values.

**Supplementary Figure 3**

**Supplementary Figure 3**

a) Western blot analysis of transfected Flag-Glt-1 expression in primary striatal LRRK2 WT and G2019S astrocytes using an anti-Flag-HRP antibody; b) Relative quantification of band intensity was performed using ImageJ and normalized to β-actin; experiment performed in triple; c) Representative epifluorescence images of Glt-1 clusters in Flag-Glt-1 transfected primary striatal astrocytes from LRRK2 WT (untreated or treated with TPA) and from LRRK2 G2019S astrocytes. Scale bars 5 μm; insets 1 μm. d) Frequency distribution of Glt-1 cluster diameter in untreated LRRK2 WT (grey bars); e) Frequency distribution of Glt-1 cluster diameter in LRRK2 G2019S (blue bars); f) Frequency distribution of Glt-1 cluster diameter in LRRK2 WT treated with TPA (d; red bars); n=5 cells analyzed for each group. Statistical analysis in b was performed using Mann-Whitney test.

**Supplementary Figure 4**

**Supplementary Figure 4**

a) Orthogonal z-stack projections of LRRK2 G2019S astrocytes co-transfected with Flag-Glt-1 (red) and GFP-Lamp1, GFP-Rab11 or GFP-Rab4 (green). Scale bar 20 μm; b) Quantitative analysis of the Pearson’s Correlation Coefficient of Glt-1 colocalizing with the Rab4-positive vesicles in LRRK2 WT and G2019S astrocytes under basal conditions or upon MLi-2 application; at least n=6 cells analyzed for each group; c) Representative confocal microscopy images of LRRK2 WT and G2019S primary striatal astrocytes transfected with GFP-Rab4 and Flag-Glt-1. Scale bars: 2 μm; d,e) Quantification of Rab4-positive vesicle number and area; at least n=9 cells analyzed for each group and experiments performed at least in triple; f) Representative confocal (left panel), electron microscopy (middle panel) and merge (right panel) image of a LRRK2 WT primary astrocyte transfected with Rab4-GFP (green). The image reveals the co-localization of early endosomal structures identified by transmission microscopy with the GFP-Rab4-positive vesicles identified by confocal microscopy; scale bar: 1 μm. Statistical analysis in b was performed using Two-way ANOVA test (Treatment F=15.79 and P=0.004; Genotypes F=16.38 and P =0.004) followed by Tukey's multiple comparisons test. Statistical analysis in d, e was performed using Unpaired t-tests.

**Supplementary Figure 5**

**Supplementary Figure 5**

a,b) Frequency distribution of Rab4-positive vesicles area in LRRK2 WT and LRRK2 G2019S astrocytes. At least n=6 cells analyzed for each group and experiments performed at least in triple.

**Supplementary Figure 6**

**Supplementary Figure 6**

a) Quantification of Glt-1 IntDen in the Rab4-positive vesicles in LRRK2 WT astrocytes under basal conditions (n=13 cells) or upon MG132 (n=13 cells) or Bafilomycin application (n=12 cells); b) Quantification of Glt-1 IntDen in the endogenous Lamp1-positive structures in LRRK2 WT astrocytes under basal conditions (n=13 cells) or upon MG132 (n=13 cells) or Bafilomycin (n=12 cells) application; experiments performed at least in triple; c) Quantitative analysis of Glt-1 IntDen in LRRK2 WT astrocytes, under basal condition or after application of MG132 or Bafilomycin A1 (n=13 cells for untreated LRRK2 WT, n=13 cells for LRRK2 WT+MG132 and n=12 LRRK2 WT+Bafilomycin; experiments performed at least in triple; Statistical analysis in a-c was performed using One-way ANOVA test (a: F=16.06 and P<0.0001; b: F=7.79 and P=0.001; c:F=8.76 and P=0.0008) followed by Tukey's multiple comparisons test.
